# Exome sequencing identifies a novel missense variant in *RRM2B *associated with autosomal recessive progressive external ophthalmoplegia

**DOI:** 10.1186/gb-2011-12-9-r92

**Published:** 2011-09-28

**Authors:** Atsushi Takata, Maiko Kato, Masayuki Nakamura, Takeo Yoshikawa, Shigenobu Kanba, Akira Sano, Tadafumi Kato

**Affiliations:** 1 Laboratory for Molecular Dynamics of Mental Disorders, RIKEN Brain Science Institute, 2-1 Hirosawa, Wako-shi, Saitama 351-0198, Japan; 2 Department of Neuropsychiatry, Graduate School of Medical Sciences, Kyushu University, 3-1-1 Maidashi, Higashi-ku, Fukuoka 812-8582, Japan; 3 Laboratory for Molecular Psychiatry, RIKEN Brain Science Institute, 2-1 Hirosawa, Wako-shi, Saitama 351-0198, Japan; 4 Department of Psychiatry, Kagoshima University Graduate School of Medical and Dental Sciences, 8-35-1 Sakuragaoka, Kagoshima-shi, Kagoshima 890-8520, Japan

## Abstract

**Background:**

Whole-exome sequencing using next-generation technologies has been previously demonstrated to be able to detect rare disease-causing variants. Progressive external ophthalmoplegia (PEO) is an inherited mitochondrial disease that follows either autosomal dominant or recessive forms of inheritance (adPEO or arPEO). AdPEO is a genetically heterogeneous disease and several genes, including *POLG1 *and *C10orf2*/Twinkle, have been identified as responsible genes. On the other hand, *POLG1 *was the only established gene causing arPEO with mitochondrial DNA deletions. We previously reported a case of PEO with unidentified genetic etiology. The patient was born of a first-cousin marriage. Therefore, the recessive form of inheritance was suspected.

**Results:**

To identify the disease-causing variant in this patient, we subjected the patient's DNA to whole-exome sequencing and narrowed down the candidate variants using public data and runs of homozygosity analysis. A total of 35 novel, putatively functional variants were detected in the homozygous segments. When we sorted these variants by the conservation score, a novel missense variant in *RRM2B*, whose heterozygous rare variant had been known to cause adPEO, was ranked at the top. The list of novel, putatively functional variants did not contain any other variant in genes encoding mitochondrial proteins registered in MitoCarta.

**Conclusions:**

Exome sequencing efficiently and effectively identified a novel, homozygous missense variant in *RRM2B*, which was strongly suggested to be causative for arPEO. The findings in this study indicate arPEO to be a genetically heterogeneous disorder, as is the case for adPEO.

## Background

Massively parallel sequencing, also known as next generation-sequencing, is a revolutionary technology that enables us to obtain large amounts of genomic sequence information in an incomparably more rapid and less expensive manner than before [1]. This technology is applicable for various investigations, including resequencing of full genomes or more targeted parts thereof for discovery of genomic variations, genome-wide mapping of structural rearrangements, transcriptome sequencing, genome-wide epigenetic analysis, metagenomic sequencing, and so on [2]. Whole-genome and whole-exome (sequences of all protein-coding regions) resequencing aiming at identification of causative variants for rare, inherited diseases is one of these applications, and have demonstrated their efficiency and effectiveness (reviewed in [3]).

Previously, we reported a patient who had been born of a first-cousin marriage and was suspected to be affected by inherited progressive external ophthalmoplegia (PEO) [4]. Inherited PEO is a form of mitochondrial disease that follows either autosomal dominant or recessive forms of inheritance (adPEO (MIM 157640; 609283; 609286; 610131, 613077) or arPEO (MIM 258450)). The characteristic findings of inherited PEOs are multiple mitochondrial DNA (mtDNA) deletions and ragged red fibers in the muscle biopsy [5]. Typical clinical symptoms are bilateral ptosis and paralysis of the extraocular muscle. Other symptoms include exercise intolerance, cataracts, hearing loss, sensory axonal neuropathy, optic atrophy, ataxia, depression, hypogonadism, and Parkinsonism [6-10].

In the present case, the recessive form of inheritance was suspected because of the patient's family history. However, no pathogenic variant in *POLG1 *(MIM 174763), which encodes a mitochondrial DNA polymerase and was the only established gene whose variants were known to cause arPEO so far, was identified [4].

The proband in this study was the only child and the available genetic information from family members was limited. Therefore, it was almost impossible to identify the causative variant using linkage analysis. On the other hand, exome sequencing using a next-generation sequencer has demonstrated its utility to detect causative variants of rare disease using a small number of samples, especially in the case of consanguineous family. Here, we performed exome sequencing in combination with runs of homozygosity (ROH) analysis in order to identify the causative variant in this patient.

## Results

### Exome sequencing identifies a novel, homozygous missense variant in *RRM2B*

A total of 3.2 Gb of sequence was generated from one lane of sequencing using the Illumina Genome Analyzer II (Illumina, San Diego, CA, USA). The proportion of the targeted exome covered at 1×, 5× and 10× was 96.3%, 88.0% and 78.3%, respectively. The mean coverage was 37.2×. A total of 19, 215 variants were detected in the coding regions defined by RefSeq Gene [11] and their flanking splice sites. The number of detected coding variants does not deviated greatly from that in previous reports [3,12]. After removing variants registered on the public database of sequence variants (dbSNP, build 130) or found in eight exomes of HapMap individuals [12] or the exome of a single, healthy, unrelated Japanese individual, which was analyzed in the same run of Illumina Genome Analyzer II sequencing, 1, 336 variants remained. Among these, 592 variants, including 141 homozygous ones, were functional (nonsense, missense, frameshift or splice site). Next, we performed ROH analysis to narrow down the candidate regions, using the base calling data on single nucleotide variants in this patient. To enhance the accuracy of the variant calling used for this analysis, 1) only the data of single nucleotide variants were used and insertion/deletion variants were excluded because of lower reliability of the detection of insertion/deletion variants [13], 2) variants called with coverage less than 8× were excluded, 3) variants called with a coverage of more than 100× were excluded because genomic regions that are known to be duplicated or have similar sequences such as pseudogenes tend to be read with high coverage. Because the primary aim of this analysis was not to evaluate ROH segments precisely, but to narrow down the list of candidate variants without overlooking the causative variant, we used relaxed criteria of ROH segments. The total size of ROH regions was 992 Mb (about 32% of the genome), which was significantly larger than the expected total size of ROH segments in an offspring born from a first cousin marriage (one-eighth of the genome). A total of 35 novel and functional variants in 33 genes were identified in ROH segments. A summary of the filtering strategy is given in Table 1.

**Table 1:** Summary of the filtering to narrow down the candidates for the causal variant

Criteria for the filtering	Number of remaining variants
Coding variants	19, 215
Not in dbSNP130	2, 015
Not in eight HapMap exomes [12]	1, 833
Not in in-house data of a healthy Japanese individual	1, 336
Functional (missense, nonsense, frameshift and splice site)	592
In run-of-homozygosity regions	35 (in 33 genes)

The filtering was performed using the listed criteria in descending order.

When we sorted these listed variants by a conservation score (phyloP score) to identify those that were most likely to be functional, a novel missense variant in *RRM2B *(g.341G > A, p.P33S), whose rare, heterozygous variant had been known to cause adPEO, was ranked at the top (Table 2).

**Table 2:** List of novel and functional variants in run-of-homozygosity regions

Chromosome	Position	Reference allele	Variant allele	Variant calling/coverage	Gene	Amino acid change	PhyloP score
8	103313660	G	A	58/58	*RRM2B*	Pro33Ser	6.741
1	39620317	G	A	5/7*	*MACF1*	Arg2523Gln; Arg3025Gln	5.329
4	107449465	A	C	63/63	*MGC16169*	Asn34Lys	5.199
22	15980313	C	T	5/5*	*LOC100287323*	Val569Ile	4.997
11	64117795	G	A	4/4*	*SLC22A12*	Trp37Stp; Trp258Stp	4.945
10	29010439	G	C	24/24	*BAMBI*	Gly108Ala	4.878
20	49482400	G	A	4/4*	*NFATC2*	Ala778Val	4.437
1	238437608	C	T	10/12	*FMN2*	Pro1101Leu	3.804
1	85362528	T	-	65/69	*WDR63*	Splice site	3.503
3	99094433	A	G	24/34	*DKFZp667G2110*	Lys546Glu	3.299
3	336547	T	G	23/23	*CHL1*	Ser30Ala	3.014
3	46595758	C	G	27/40*	*LRRC2*	Arg41Gly	2.522
4	169335658	A	C	9/13*	*ANXA10*	Thr193Pro	2.257
5	140538797	C	T	127/127	*PCDHB8*	Thr333Ile	2.011

Variants with PhyloP score > 2 are listed. Asterisks indicate variants with coverage < 8× or a variant calling/coverage ratio < 0.7; the reliability of these variant calls is generally lower than that of the others.

**Figure 1:**
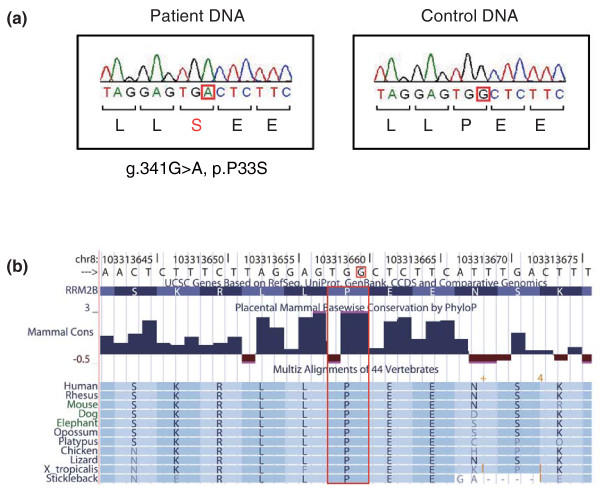
**The identified disease-associated variant in *RRM2B***. **(a) **Partial sequence of *RRM2B *in the patient's DNA (left) and control DNA (right). Red squares indicate the base position of the g.341G > A, p.P33S variant. **(b) **The substituted amino acid residue (red box) is highly conserved across 44 vertebrate species (from the UCSC genome browser [31]).

The existence of the *RRM2B *variant in the patient's DNA was confirmed by Sanger sequencing (Figure 1a). As expected, each of the parents had this variant in the heterozygous state. This variant changes an amino acid residue that is highly conserved across 44 vertebrates (Figure 1b). Among 359 control subjects (718 chromosomes) of Japanese origin, one subject carried this variant in the heterozygous state.

### Exclusion of other variants that could cause PEO

In the list of 35 novel and functional variants in the ROH segments, no other variants in genes encoding mitochondrial proteins were registered in Human MitoCarta [14]. We could not find any pathogenic mutations in other genes known to cause mitochondrial diseases with multiple mtDNA deletions (*POLG1*, *POLG2 *(MIM 604983), *C10orf2 *(MIM 606075), *SLC25A4 *(MIM 103220), *OPA1 *(MIM 605290), *TYMP *(MIM 131222) and *WFS1 *(MIM 606201)) in exome analysis, as was observed in a previous study using Sanger sequencing [4]. Although the mtDNA sequence was not targeted by the SureSelect Human All Exon Kit (Agilent, Santa Clara, CA, USA), 16, 558 of 16, 568 (99.9%) bases in mtDNA were read four or more times due to its higher copy number than nuclear DNA, and no known pathogenic variant was found. Because of the family history of the patient, we suspected that his disease was caused by a recessive mutation. However, there was another possibility that *de novo *variants affect him in a dominant manner. To test this possibility, we investigated whether he had *de novo *variants that could explain his symptoms. In the list of 592 novel and putatively functional variants, there were 26 heterozygous variants in genes registered in MitoCarta. Among them, five variants were not found in dbSNP132 or 1000 Genome Project data [15] (SNP calls released in June 2011), and were located at conserved base positions (phyloP score > 2). By performing Sanger sequencing, we confirmed that all of these variants were not *de novo*, but inherited from either of his healthy parents or found as a false positive (Table 3).

**Table 3:** List of novel, putatively functional and heterozygous variants in mitochondrial genes

Chromosome	Position	Reference allele	Variant allele	Variant calling/coverage	Gene	Amino acid change	PhyloP score	Inheritance
7	30615756	G	C	36/69	*GARS*	Asp256His	6.494	Paternally inherited
10	104476790	T	T	14/30	*SFXN2*	Leu73Pro	4.906	Maternally inherited
7	100670236	C	C	20/51	*FIS1*	Ala90Pro	3.824	Maternally inherited
11	47620527	A	A	3/8	*MTCH2*	Tyr23His	3.680	Not confirmed in Sanger sequencing
1	10286026	C	G	22/46	*KIF1B*	Ile732Met	3.092	Maternally inherited

Variants with PhyloP score > 2 are listed.

### Evaluation of the amount of mtDNA

**Figure 2:**
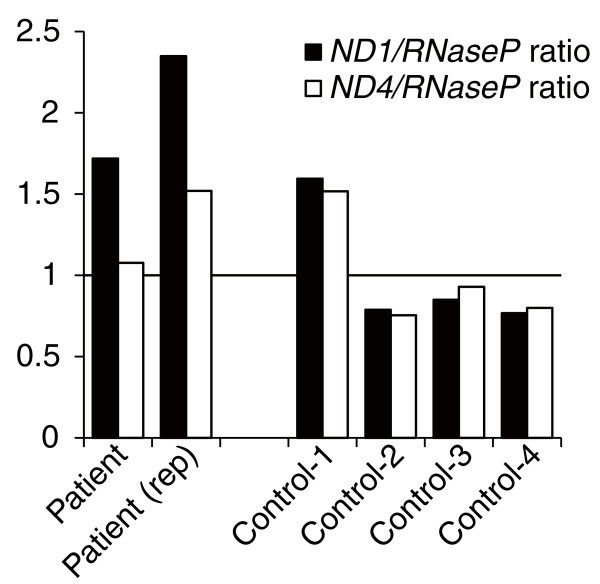
**Relative amounts of mtDNA in skeletal muscle tissues from the patient and four control subjects**. *ND1*/*RNaseP *and *ND4*/*RNaseP *ratios calculated by real-time quantitative PCR were used to evaluate mtDNA levels. The mtDNA level in the patient was comparable to those of controls. Values are relative to the average of all four controls.

The mtDNA copy number relative to nuclear DNA in the patient's skeletal muscle was not decreased, but rather increased (Figure 2). As expected, the *ND4*/*RNaseP *ratio was lower than the *ND1*/*RNaseP *ratio in the patient, which suggests increased levels of mtDNA deletions that include the *ND4 *region, such as the 4, 977-bp common mtDNA deletion [16]. This result indicated that the clinical manifestation in the present patient was not due to mtDNA depletion.

## Discussion

In this study, we subjected DNA from a PEO patient with unidentified genetic etiology to exome sequencing and detected a novel, homozygous missense variant in *RRM2B*. *RRM2B *encodes p53-inducible ribonucleotide reductase small subunit 2-like protein (p53R2) and this protein plays an essential role in the maintenance of mtDNA by reducing ribonucleotides in the cytosol [17], as is indicated by the fact that rare variants in this gene cause various forms of mitochondrial diseases characterized by mtDNA depletion and deletions. To our knowledge, 15 cases of mitochondrial depletion syndrome (MIM 612075) from 11 families [18-22] and one sporadic case of mitochondrial neurogastrointestinal encephalopathy [23] (MIM 603041) associated with homozygous or compound heterozygous rare variants in *RRM2B *have been reported. More recently, two families with adPEO due to a heterozygous nonsense variant were described [24]. In the screening of *RRM2B *variants in 50 mitochondrial disease patients without causative variants in *POLG1 *and *C10orf2*, one Kearns-Sayre syndrome (MIM 530000) patient who carried two different novel missense variants and one PEO patient who carried an in-frame deletion were identified [25].

The clinical symptoms and findings in the muscle biopsy of our case were typical for Mendelian-inherited PEO. No members of his maternal family have shown any neuromuscular symptoms, suggesting that the mtDNA deletions of the patient were not maternally inherited. Real-time quantitative PCR analysis revealed that there was no mtDNA depletion. We did not observe gastrointestinal dysmotility, cardiac conduction abnormalities, pancreatic dysfunction and sensory ataxic neuropathy, which are characteristic symptoms for other mitochondrial diseases associated with mtDNA deletions, namely mitochondrial neurogastrointestinal encephalopathy, Kearns-Sayre syndrome, Pearson syndrome, and sensory ataxic neuropathy, dysarthria, and ophthalmoparesis (MIM 607459), respectively. Therefore, this patient was diagnosed as having arPEO caused by a homozygous missense variant of *RRM2B*.

Before this study, *POLG1 *had been the only established gene responsible for arPEO, while adPEO is a genetically heterogeneous disease, caused by rare variants in *POLG1, POLG2, C10orf2, SLC25A4, OPA1 *and *RRM2B*. The results of this study identifying the second responsible gene for arPEO indicate that arPEO is also a genetically heterogeneous disease, as is the case for adPEO.

The symptoms observed in this patient included major depressive episodes. Frequent comorbidity of mood disorders in patients of mitochondrial disease has been generally recognized [26] and several lines of evidences have supported the possible involvement of mitochondrial dysfunctions in the pathophysiology of mood disorders [27]. So far, rare variants of *POLG1*, *C10orf2 *and *SLC25A4 *have been reported in inherited PEO pedigrees with frequent comorbidity of mood disorders [28]. Given the typical symptoms of major depressive disorder in the present case, *RRM2B *should be added to the list of genes causal for PEO associated with mood disorders.

The identified P33S variant changes an amino acid residue highly conserved among vertebrates. The amino-terminal region of p53R2, in which this altered amino acid is located, is suggested to be crucial for interaction with p21 protein. p53R2 may contribute to DNA repair in cooperation with p21 [29]. In its amino-terminal region, the homozygous p.R41P variant was detected in a mitochondrial depletion syndrome case [21]. On the other hand, other pathogenic missense variants have been located in various sites of p53R2, including those involved in iron-binding [18,20], those putatively crucial for homodimerization of p53R2 [21,23] or heterotetramerization with the RRM1 (ribonucleoside-diphosphate reductase large subunit) homodimer [18,22], and so on. The relationships between clinical phenotypes and the properties of variants, as well as their underlying mechanisms, should be the subject of further investigations.

## Conclusions

In this study, we describe a homozygous missense variant in *RRM2B *that is strongly suggested to cause arPEO. We were not only able to identify the disease-associated variant, but could also exclude other candidates (that is, variants in known PEO-related genes such as *POLG1*, other mitochondrial genes in nucleic DNA and mtDNA) using data from single exome sequencing. This result further demonstrates the efficiency and effectiveness of exome sequencing to detect causative variants of rare, inherited, and genetically heterogeneous diseases.

## Materials and methods

### Clinical information of the patient

The detailed clinical history, family history and laboratory data of the studied subject are described elsewhere [4]. Briefly, a 43-year-old man presented with hearing loss, bilateral ptosis, external ophthalmoplegia and muscle weakness. Examinations revealed the existence of pigmentary degeneration of the retina and gonadal atrophy. The initial symptom of progressive hearing loss began at age 16 years. Depressive mood, anxiety and hypochondriacal complaints were observed in his clinical course. His parents were first cousins, he had no siblings, and no other member of his family has a known history of neurological illness. In the muscle biopsy, marked variation of muscle fiber size, ragged red fibers, COX-negative fibers and multiple mtDNA deletions were detected. According to his clinical history, family history and laboratory data, arPEO was suspected.

The present study conformed to the Declaration of Helsinki, and was approved by the RIKEN Wako Institute Ethics Committee I, as well as the ethics committees of Kagoshima University Graduate School of Medical and Dental Sciences and other participating institutes. Written informed consent was obtained from every subject.

### Exome sequencing and data analysis

Total DNA was obtained from peripheral blood of the patient using standard protocols. Total DNA (3 μg) was sheared into approximately 300-bp fragments using a Covaris sonicator (Covaris, Woburn, MA, USA). A paired-end exome library for Illumina sequencing was prepared using the SureSelect Human All Exon Kit (Agilent) following the manufacturer's instructions. Massively parallel sequencing was performed using one lane of the Genome Analyzer II (Illumina) at RIKEN Omics Science Center by the Life Science Accelerator system. Base calling was performed by the Illumina pipeline with default parameters. Obtained reads were mapped against the human reference genome (UCSC hg18/GRCh36) using CLC Genomics Workbench v4.0.2 software (CLC Bio, Aarhus, Denmark) with default parameters. Variant calling was performed using the SNP and DIP detection tools in CLC Genomics Workbench v4.0.2 with default parameters. Analysis of ROH was performed using PLINK software v1.0.7 [30]. The primary aim of this analysis was not to evaluate ROH segments precisely, but to narrow down the list of candidate variants without overlooking the causative variant. Therefore, we used relatively small (1, 000 kb) sliding windows for ROH segments, did not consider local blocks of linkage disequilibrium in the Japanese population, and did not exclude the data of variants whose frequency was not registered in dbSNP; those variants might not be polymorphic in the Japanese population and possibly contributed to extend the length of ROH. Conservation information for the variants among 44 vertebrate species (phyloP score) was collected from the UCSC genome browser [31].

### Sanger sequencing

Sanger sequencing of PCR amplicons was performed to confirm the detected disease-associated variant using a 3730 × L DNA Analyser (Applied Biosystems, Foster City, CA, USA). The primers used were: forward, 5'-AGGCAGACAGGCTCTCAAAC-3'; reverse, 5'-GGCAGAATTAGATGCCATTG-3'.

### Real-time quantitative PCR

The amount of nuclear DNA and mtDNA in the skeletal muscle of the patient and four age- and sex-matched controls (all males aged 39 to 48 years) was evaluated by real-time quantitative PCR analysis according to the previously validated methods [32]. Briefly, copy numbers of *RNaseP *(for nuclear DNA), *ND1 *and *ND4 *(for mtDNA) were evaluated using the TaqMan method (Applied Biosystems). Analysis of the patient's tissue was performed in two independent reactions, and each experiment was triplicated. *ND1*/*RNaseP *and *ND4*/*RNaseP *ratios were calculated as 2^[Ct(*RNaseP*)-Ct(each gene)]^.

### Data accessibility

The sequence data from this study have been submitted to dbGaP [33] (study accession [phs000392.v1.p1]).

## Abbreviations

adPEO: autosomal dominant progressive external ophthalmoplegia; arPEO: autosomal recessive progressive external ophthalmoplegia; mtDNA: mitochondrial DNA; PEO: progressive external ophthalmoplegia; ROH: runs of homozygosity.

## Competing interests

The authors declare that they have no competing interests.

## Authors' contributions

AT and TK designed the study and drafted the manuscript. AT performed data analysis and molecular experiments. MK, MN and AS performed clinical assessment. MK, MN, TY and AS provided materials for experiments. TY, SK, AS and TK coordinated the study and performed critical revision of the manuscript. All authors read and approved the final manuscript.
